# Pediatric ocular tuberculosis: a case report of complex clinical manifestations and its successful management

**DOI:** 10.11604/pamj.2024.49.68.45595

**Published:** 2024-11-08

**Authors:** Affannul Hakim, Evelyn Komaratih, Ismi Zuhria

**Affiliations:** 1 Department of Ophthalmology, Dr. Soetomo General Academic Hospital, Surabaya, Indonesia,; 2 Department of Ophthalmology, Faculty of Medicine, *Universitas Airlangga*, Surabaya, Indonesia

**Keywords:** Infection, tuberculosis, ocular tuberculosis, children, case report

## Abstract

Ocular tuberculosis in children poses significant visual risks and exhibits distinct characteristics compared to adults, necessitating careful diagnosis and management. This case illustration presents a 10-year-old girl with intermittent redness in her left eye. Despite initial treatment, her condition worsened, leading to blurred vision. She was diagnosed with ocular tuberculosis complicated by phlyctenular conjunctivitis, interstitial keratitis, diffuse anterior scleritis, anterior uveitis, and neuroretinitis. Antituberculosis therapy was initiated alongside corticosteroids and neuroprotective treatment. The patient showed significant improvement within one month and achieved full resolution after two months of treatment. Ocular tuberculosis in children, it often accompanies extrapulmonary tuberculosis and can lead to significant complications. Additionally, children tend to exhibit a heightened inflammatory response to ocular tuberculosis, necessitating more aggressive corticosteroid therapy to manage the condition effectively.

## Introduction

Children are particularly vulnerable to tuberculosis (TB), with pediatric cases comprising about 10% of all reported TB cases. In 2015, approximately 1 million new TB cases were estimated among children worldwide, resulting in about 210,000 deaths in this population. Tuberculosis infection requires inhaling approximately 5 to 200 bacilli, and the immune response plays a crucial role, especially in organs with high oxygen tension, such as the lungs, kidneys, bones, meninges, eyes, and choroid [1,2]. Extrapulmonary TB can occur in up to 20% of cases, with ocular involvement reported in 3% to 5% of patients, potentially leading to significant visual morbidity. In children, ocular tuberculosis exhibits distinct characteristics compared to adults [3]. This case report highlights the clinical manifestations of ocular tuberculosis in a child and demonstrates a remarkable positive response to the treatment provided.

## Patient and observation

**Figure 1:**
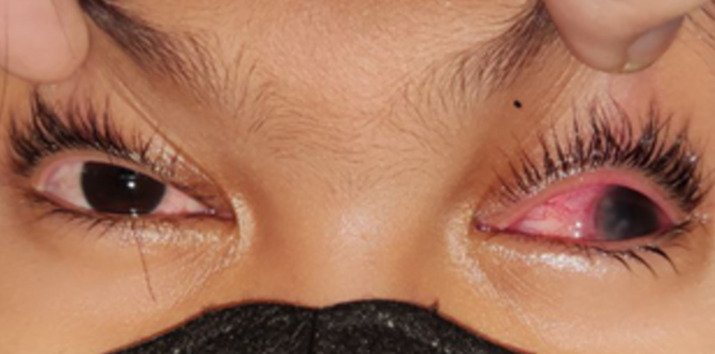
the patient's left eye appears red, accompanied by photophobia and eyelid spasm

**Patient information:** a 10-year-old girl presented with complaints of intermittent redness in her left eye over the past month. The redness was associated with a gritty sensation, pain, and photophobia (Figure 1). Two weeks ago, a clear bump appeared at the edge of her cornea, which resolved after one week. Following that, the patient noticed her eye discharging clear fluid, resembling tears. Despite the redness continuing intermittently, one week prior to the visit, her vision became blurry. One day before this visit, another white bump appeared at the edge of her cornea, again without discomfort or pain.

**Figure 2:**
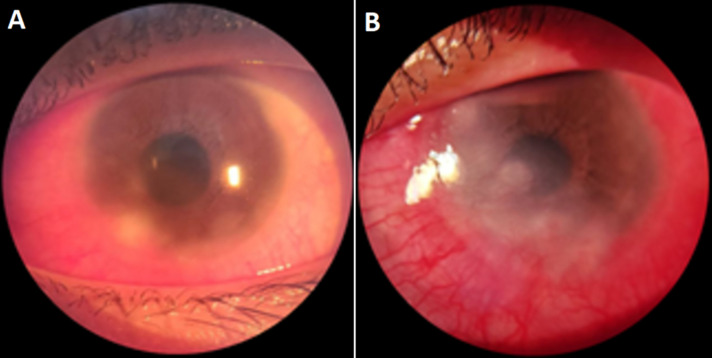
anterior segment examination of the patient's left eye at the initial visit (A) and one month later (B)

**Clinical findings:** visual acuity of the right eye was 6/6 and the left eye was 6/9, intraocular pressure (IOP) was 12 mmHg in her right eye and 11 mmHg in her left eye. On anterior segment examination, there is eyelid spasm of both eyes and a 3 x 3 mm nodule was observed at the 7 o´clock position on the corneal margin of the left eye, suggesting phlyctenule (Figure 2A). The initial diagnosis was phlyctenular conjunctivitis, and she was prescribed prednisolone, atropine, ofloxacin, artificial tears, and chloramphenicol ointment for the left eye.

**Figure 3:**
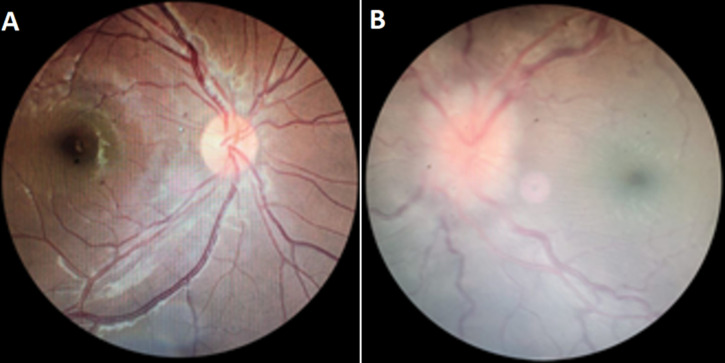
posterior segment examination of the patient's right eye (A) and left eye (B)

She returned for follow-up twice within the following month due to worsening redness and blurred vision in the left eye. Despite ongoing treatment, her condition continued to deteriorate, with no significant improvements were noted. In the fourth follow-up, one month after the initial visit, the patient returned with significantly worsened symptoms. The visual acuity of the left eye had deteriorated to counting fingers at three meters, and the intraocular pressure (IOP) had dropped to 6 mmHg. On anterior segment examination of the left eye, there was an eyelid spasm in both eyes, along with marked inferior conjunctival and ciliary injection. Phenylephrine testing showed partial resolution of hyperemia. The cornea appeared cloudy with anterior and stromal infiltrates in the inferior area, posterior edema in the superior region, fibrosis, neovascularization, and mutton-fat keratic precipitate. The anterior chamber exhibited flare and cells. (Figure 2B). Posterior segment examination of the right eye was normal, while in the left eye, it revealed a positive fundus reflex, with a blurred and hyperemic optic disc margin. The inferior retina appeared pale, accompanied by vascular tortuosity (Figure 3A,B).

**Figure 4:**
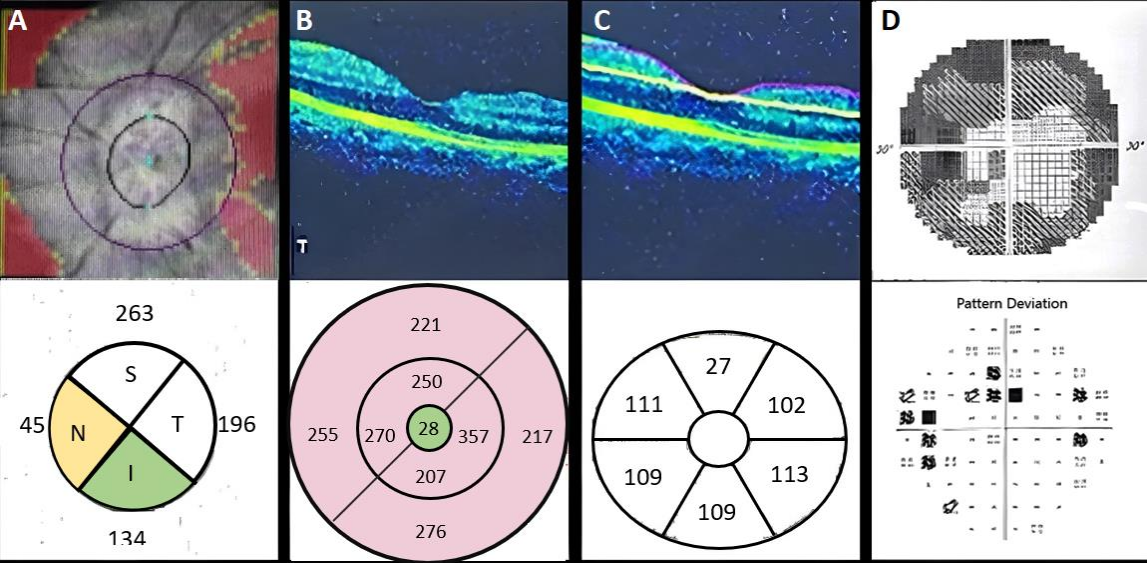
auxiliary examination results of the left eye: (A) optical coherence tomography (OCT) optic nerve head; (B) OCT macula ILM-RPE complex; (C) OCT macula GCL-IPL complex; (D) Humphrey field analyzer

**Diagnostic assessment:** left eye´s optical coherence tomography (OCT) of the optic nerve showed thickening of the retinal nerve fiber layer in the superior and temporal quadrants (Figure 4A). OCT of the macula showed thickening of the ILM-RPE (Figure 4B) and GCL-IPL complex (Figure 4C). A Humphrey visual field test indicated paracentral scotomas on the left eye (Figure 4D), and a whole-body exam showed no lesions or lymphadenopathy.

The patient was referred to the pediatric department for evaluation of tuberculosis (TB) and other autoimmune conditions. A positive Mantoux test, along with a history of contact with a neighbor diagnosed with TB, led the pediatric department to diagnose the patient with extrapulmonary TB.

**Diagnosis:** we diagnosed the patient with Ocular TB, complicated by phlyctenular conjunctivitis, interstitial keratitis, diffuse anterior scleritis, anterior uveitis, and neuroretinitis in the left eye.

**Therapeutic interventions:** the patient was treated with topical prednisolone eye drops (3 times daily for the left eye), atropine eye drops (twice daily for the left eye), artificial tears (6 times daily), and chloramphenicol eye ointment (3 times daily for the left eye). Additionally, systemic treatment included methylprednisolone (16 mg, twice daily), neurotropic medication (once daily), and a fixed-dose combination of intensive-phase anti-tuberculosis drugs (3 tablets once daily).

**Figure 5:**
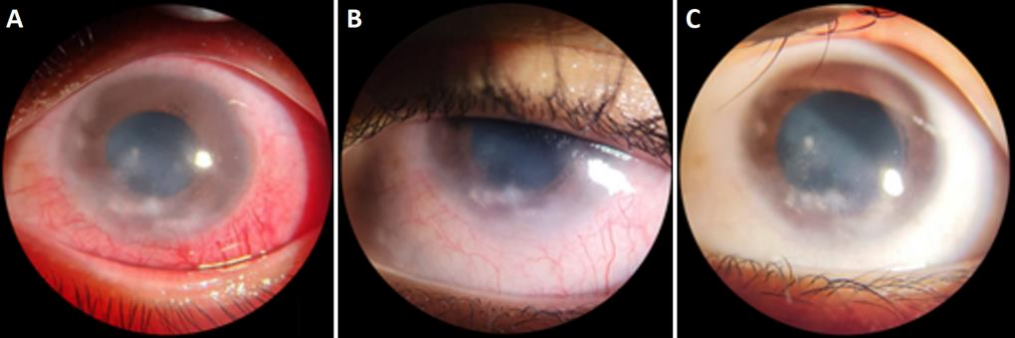
(A) anterior segment examination of the left eye at 2-week follow-up; (B) 1-month follow-up; (C) and 2-month follow-up after anti-tuberculosis therapy

**Follow-up and outcome of interventions:** in the follow-up visits over the next two weeks and one month later, the patient demonstrated significant improvement in her condition. During the two-week follow-up (Figure 5A), she reported reduced redness in her left eye, with visual acuity improving to 6/45 and IOP recorded at 9 mmHg. By the one-month follow-up (Figure 5B), her visual acuity further improved to 6/7, and her IOP was stable at 13 mmHg. Examination findings revealed decreased conjunctival and ciliary injection, reduced corneal cloudiness, diminished vascularization, fewer infiltrates, and no flare or cells in the anterior chamber indicating a positive response to treatment.

After two months of tuberculosis treatment, her condition showed remarkable improvement. During the follow-up examination, she reported that her eye was no longer red and her vision had returned to clarity. The eye examination revealed a visual acuity of 6/6 and a stable IOP of 12 mmHg in the left eye. Examination of the anterior segment indicated that the conjunctiva was clear, with no signs of conjunctival or ciliary injection. The cornea also appeared clear, with no infiltrates or edema; however, a small 3 x 1 mm nebula was noted in the inferior area. All other findings were normal (Figure 5C). The treatment plan included continuing the use of artificial tears six times a day and transitioning the anti-tuberculosis therapy to the maintenance phase.

**Patient perspective:** “at first, I was really scared when my eye became red and blurry. After starting the treatment, I was gradually relieved to see clearly now and feel much better”.

**Informed consent:** the patient and her parents were informed of the case reported and agreed that it should be published for the benefit of people and medicine.

## Discussion

Ocular tuberculosis, a manifestation of extrapulmonary tuberculosis, can affect various eye structures and present unilaterally or bilaterally. Its clinical manifestations vary widely and often mimic other ocular disorders. In children, ocular tuberculosis resembles adult cases but is more frequently linked to extrapulmonary tuberculosis, with potential complications including conjunctivitis, interstitial keratitis, scleritis, choroiditis, and optic nerve inflammation [4].

In this case, the patient initially experienced recurrent phlyctenular conjunctivitis, which eventually ruptured. Despite treatment, her condition worsened, progressing to interstitial keratitis and diffuse anterior scleritis. Literature indicates that ocular tuberculosis can cause conjunctival ulceration, nodular lesions, and hypertrophic papillary lesions, with keratoconjunctivitis phlyctenular often being a delayed hypersensitivity reaction to mycobacterial antigens [5]. Interstitial keratitis may occur alone or alongside scleritis and uveitis, frequently linked to tubercular antigens in the aqueous humor. Scleritis can result from direct scleral invasion by *Mycobacterium tuberculosis* or an immune response to its antigens. Symptoms may be localized or diffuse, usually marked by nodular lesions. This is particularly relevant in our case, as other studies have shown that children exhibit a heightened inflammatory response to ocular tuberculosis infection and its associated antigens compared to adults [4-6].

In our case, the patient exhibited anterior uveitis, characterized by keratic precipitates, cells, and flare in the anterior chamber. The uvea, due to its rich vascular supply and high oxygen content, is particularly susceptible to tuberculosis infection. Recent evidence indicates that the infection can spread hematogenously to both the anterior and posterior chambers, leading to acute or chronic granulomatous anterior uveitis, often featuring iris granulomas, mutton-fat keratic precipitates, and posterior synechiae. While ocular tuberculosis typically involves the ciliary body and choroid, neither area was affected in this case. The involvement of the ciliary body can result in intermediate uveitis, marked by small gray nodules that may enlarge and vascularize if untreated. In the choroid, ocular tuberculosis can lead to posterior uveitis, categorized into choroidal tubercles, choroidal tuberculomas, and serpiginous-like choroiditis [7].

The posterior segment examination in our case showed swelling of the optic nerve head and retina, indicating neuroretinitis. Optic nerve involvement is a common complication of tuberculosis and can result from direct infection, spread from the choroid, hematogenous dissemination, or hypersensitivity to the infectious agent. Recent multicenter studies have found that the most common forms of optic nerve involvement are papillitis (51.6%), neuroretinitis (14.5%), and optic nerve tubercles (11.3%). Swelling of the optic nerve head can arise from various causes, including tuberculous posterior scleritis or central nervous system tuberculosis, particularly tuberculous meningitis, which may lead to hydrocephalus, optico-chiasmatic arachnoiditis, and optico-chiasmatic tuberculoma [8].

Treatment for ocular tuberculosis aligns with strategies for pulmonary or extrapulmonary tuberculosis, typically resulting in improved ocular inflammation with systemic therapy. The Centres for Disease Control and Prevention (CDC) recommends an initial two-month regimen of four drugs (isoniazid, rifampicin, pyrazinamide, and ethambutol), followed by a four-month continuation phase with isoniazid and rifampicin, totaling six months of treatment. This duration may extend to 9-12 months based on patient response. If there is a poor therapeutic response, reassessment is crucial, as the likelihood of ocular tuberculosis may be reduced. However, exceptions apply when intraocular tuberculosis is confirmed through culture, histopathology, or polymerase chain reaction (PCR) [9]. In our case, the patient initially showed a poor response, with increasing redness and blurriness in the eye. After starting antituberculosis therapy, significant improvement was observed within one month, with complete resolution in the left eye after two months.

We administered both topical and systemic steroids to our patient, which were crucial for managing her condition. This was especially important given the patient's manifestations, including phlyctenular conjunctivitis, interstitial keratitis, diffuse anterior scleritis, and uveitis [5,6]. These symptoms are typically due to hypersensitivity reactions to mycobacterial antigens. Low-dose corticosteroids, given over 4 to 6 weeks alongside multidrug antituberculosis therapy, have been shown to reduce ocular tissue damage from delayed-type hypersensitivity in ocular tuberculosis cases [10]. In addition to topical steroids, we also administered systemic corticosteroids in this case, as the administration of corticosteroids in children should be more aggressive due to their heightened inflammatory response compared to adults [4].

## Conclusion

Pediatric ocular tuberculosis presents significant challenges, it often features heightened inflammatory responses due to hypersensitivity to *Mycobacterium tuberculosis* antigens. A 2-month intensive antituberculosis treatment, followed by a 4-month continuation phase, has shown favorable outcomes. Aggressive steroid therapy is also recommended to manage inflammatory reactions effectively.
